# Visual Search Strategies of Soccer Players Executing a Power vs. Placement Penalty Kick

**DOI:** 10.1371/journal.pone.0115179

**Published:** 2014-12-17

**Authors:** Matthew A. Timmis, Kieran Turner, Kjell N. van Paridon

**Affiliations:** Sport and Exercise Sciences Research Group (SESRG), Department of Life Sciences, Anglia Ruskin University, Cambridge, United Kingdom; University of Bath, United Kingdom

## Abstract

**Introduction:**

When taking a soccer penalty kick, there are two distinct kicking techniques that can be adopted; a ‘power’ penalty or a ‘placement’ penalty. The current study investigated how the type of penalty kick being taken affected the kicker’s visual search strategy and where the ball hit the goal (end ball location).

**Method:**

Wearing a portable eye tracker, 12 university footballers executed 2 power and placement penalty kicks, indoors, both with and without the presence of a goalkeeper. Video cameras were used to determine initial ball velocity and end ball location.

**Results:**

When taking the power penalty, the football was kicked significantly harder and more centrally in the goal compared to the placement penalty. During the power penalty, players fixated on the football for longer and more often at the goalkeeper (and by implication the middle of the goal), whereas in the placement penalty, fixated longer at the goal, specifically the edges. Findings remained consistent irrespective of goalkeeper presence.

**Discussion/conclusion:**

Findings indicate differences in visual search strategy and end ball location as a function of type of penalty kick. When taking the placement penalty, players fixated and kicked the football to the edges of the goal in an attempt to direct the ball to an area that the goalkeeper would have difficulty reaching and saving. Fixating significantly longer on the football when taking the power compared to placement penalty indicates a greater importance of obtaining visual information from the football. This can be attributed to ensuring accurate foot-to-ball contact and subsequent generation of ball velocity. Aligning gaze and kicking the football centrally in the goal when executing the power compared to placement penalty may have been a strategy to reduce the risk of kicking wide of the goal altogether.

## Introduction

Visually exploring the environment during a game of soccer allows key features to be identified, subsequently facilitating the planning of appropriate motor responses. Previous research has shown that the visual search strategies soccer players employ when viewing the environment is dependent upon the type of task presented [1]. Indeed, varying the number of offensive and defensive players within the environment significantly influences a players’ visual search strategy [2]–[6]. Vayens et al. [2], [3] demonstrated that when the number of players in the environment increases, players change from exhibiting a low search rate with prolonged fixations to a higher search rate of shorter duration; a finding supported when comparing results from players viewing 11 vs. 11 [4], [5] and 3 vs. 3 match situations [6]. These changes in visual search strategy occur due to a greater number of potentially informative areas from which task relevant information can be extracted to inform decision making [3].

The soccer penalty kick, a relatively ‘closed’ skill [5], has been regularly used as a method of investigating the visual search strategies of both outfield players (penalty kicker) and goalkeepers. Research has shown that a goalkeeper’s visual search strategy is influenced by their ability and/or expertise (e.g. [7]–[9]) and the penalty kicker’s by the movements or presence of a goalkeeper [10], [11], and the strategy the kicker adopts (i.e. a goalkeeper dependent or independent strategy; see [11]–[13] for details). The psychological state (e.g. anxiety) also impacts kicking performance [12].

When taking a penalty kick, there are two distinct kicking techniques that can be adopted; a ‘power’ penalty or a ‘placement’ penalty. When taking a power penalty, the penalty taker adopts a technique where the football is kicked with maximum power, which subsequently generates a high ball velocity. This minimises the time the goalkeeper has to initiate a response to save the football; from the instance the football is kicked, goalkeepers typically have between 300 and 800 milliseconds to react and save a penalty [14], [15]. Alternatively, the player can take a placement penalty, adopting a technique where they aim to accurately kick the football to an area of the goal that the goalkeeper cannot easily reach (i.e. the corners of the goal). Both techniques are regularly used when taking a penalty kick [14]. Through analysing the ball flight times of 66 penalties from the German Bundesliga and European cup matches (sampled during the 1981–82 and 1982–83 seasons), Kuhn [14] demonstrated that 20% of penalties were identified as a power penalty (flight times <600 ms, ∼21 m.s^−1^) and 80% as a placement penalty (flight times ≥600 ms).

It is widely accepted that as the speed of the movement increases, the accuracy of the movement decreases (termed Fitts’ law [16]). This speed-accuracy trade-off has been evidenced during ball-kicking actions. For example, compared to when kicking a ball accurately to a specific location, kicking a ball with maximum power results in higher ball velocity [17], [18] which reduces the accuracy of the kick [19], [20]. Compared to when executing a placement penalty, the reduction in accuracy when executing a power penalty may affect the visual search behaviour of the kicker, causing them to fixate at the football for longer in order to retain some element of control/accuracy in the kick.

Whilst Kuhn [14] demonstrated that both power and placement penalties are regularly taken in soccer, no detail was provided regarding end ball location as a function of the type of penalty kick. Through descriptive analysis of 311 penalties from football leagues, world cup and European championships (precise leagues and year(s) penalties were sampled from were not indicated), Bar-Eli and Azar [21] reported that 6% of penalties missed the goal altogether: a value similarly reported by Jordet et al. [22]. Of those that were not kicked wide of the goal, 29% of kicks landed in the middle of the goal and 71% towards the edges of the goal [21]. These values were similar to those reported from the 1986 Mexican World cup [23]. However, no distinction was made between the technique used in the penalty kick.

Previous research has shown that physically executing a task results in very different visual search patterns compared to when observing a task [24]. The change in visual search pattern is likely attributed to the shift from target selection for action based on intrinsic salience to one based on task instruction [25]. Whilst there is extensive research which has monitored visual search behaviour of soccer players viewing footage presented on video screens (e.g. [2]–[6]), there is little research investigating soccer players visual search strategies executing real world activities. Indeed, to date there has been no research investigating how a power compared to placement penalty kick affects the kicker’s visual search strategy in a dynamic action; this was the aim of the present study. A secondary aim was to investigate whether the type of penalty being taken affects end ball location.

## Methods

### Participants

12 University footballers (age 20.1±1.4 years; mean ± SD) participated in the study. All had experience of playing competitive football (ranging from amateur to semi-professional) for 12.6±3.4 years. All reported regularly taking penalties for their respective clubs.

The tenants of the Declaration of Helsinki were observed and Anglia Ruskin University’s Ethical Committee approved the study. Written informed consent was obtained from each participant prior to participation.

### Apparatus

The study was conducted indoors according to The Football Association (F.A.) guidelines for 5, 6 and 7-a-side indoor football [26]. A size 4 football (Mitre super league indoor football) was placed on a penalty spot 6 metres from the centre of a goal measuring 3.66 m wide by 1.83 m high. Gym mats (0.03 m thickness) were placed in front of the goal to prevent injury to the goalkeeper when diving to save the football.

A GoPro (Hero 3, San Mateo, California) high speed video camera was placed (at a height of ∼0.3 m) perpendicular to the penalty spot (distance of 1.5 m) to record (at 200 Hz) the displacement of the football once kicked. Data recorded from the high speed camera were analysed using WinAnalyze software (Mikromak; Berlin, Germany) to calculate initial velocity of the football when kicked. Only one power and placement kick was recorded for each participant to provide an indication of ball velocity.

A Canon (Legria HD HF R28, Tokyo, Japan) video camera was positioned 10 m from the goal, behind the penalty kicker, to record (at 25 Hz) the end location of the football after the penalty had been taken (either within the goal, irrespective of whether the penalty was saved or not, or wide of the goal on the wall behind the goal). Screen shots from the video identifying the football’s end location were taken using XnView (Ver. 1.99.6; Neuvilette, France) and uploaded into Didge (Image Digitizing Software Ver. 2.30b1; Omaha, Nebraska, USA) to allow the horizontal and vertical end location of the football to be defined.

Eye movements of the penalty taker were recorded using an SMI iViewETG head mounted mobile eye tracker (SensoMotoric Instruments Inc, Warthestr; Germany, Ver. 1.0) at 30 Hz. The eye tracker contains 3 cameras built into the glasses, an infrared camera to record movements of each eye and a high definition (HD) camera (1280×960 pixel, 24 Hz) to record the visual scene. Data from the eye tracker were recorded on a mini laptop (Lenovo X220, ThinkPad, USA) with iView ETG (Ver. 2.0) recording software installed. The laptop was placed in a backpack worn by the participant during testing. A simple three point eye calibration was performed to verify point-of-gaze before each participant was tested. The calibration was checked following every third trial. The spatial resolution of the system was 0.1°, with gaze position accuracy of ±0.5°.

In order to identify where within the goal area participants were fixating during the penalty, the goal area was divided evenly into 3 sections (left, middle and right).

### Procedure

Participants initially warmed up for approximately 10 minutes prior to taking the penalties. The warm up consisted of a series of dynamic stretches, dribbling and passing drills. Participants completed the warm up wearing the eye tracker to become accustomed to the glasses and backpack. None of the participants reported that either the eye tracker or backpack inhibited range of movement.

When taking the penalties, participants were instructed to score as many goals as possible and avoid attempting to deceive the goalkeeper using strategies such as looking in one direction and kicking in the other (cf. opposite independent strategy [11]), or pausing during the run up to take the penalty. Prior to the participant placing the football on the penalty spot to take the penalty, they were instructed to take either a placement or power penalty (the goalkeeper was not aware of the instruction). When instructed to take a placement penalty, participants were asked to place the ball in an area of the goal where they thought they could score. No instruction was given pertaining to the specific area of the goal the football was to be kicked towards. In the power penalty, participants were instructed to generate as much power during the kick, whilst also attempting to score. Instructions were developed in such a way to avoid potential ironic effects impacting the experiment (see [27]). Two penalties were completed in each power and placement condition in a randomised order.

Participants were required to execute both power and placement penalties with and without the presence of a goalkeeper; data collected in a block randomised order. In the goalkeeper present condition, the goalkeeper was instructed to save as many penalties as possible. The goalkeeper was instructed (when present) to initially stand in the centre of the goal, with their arms positioned outstretched in front of them (replicating the typical position of a goalkeeper when facing a penalty kick). The goalkeeper was instructed to remain still, in the same position in the goal and not attempt to anticipate the ball direction prior to the football being kicked. Goalkeeper position was standardised throughout the experiment to minimise their influence on the direction that the penalty taker kicked the ball [11], [28]. In the no goalkeeper condition, participants were required to execute the power and placement penalties adhering to the instructions provided in the goalkeeper present condition. A no goalkeeper condition was included in the study design to ameliorate any potentially distracting effects of the goalkeeper (see [13]). The same goalkeeper was used throughout the study.

Four penalties kicks (2 power and 2 placement) were taken in each goalkeeper condition (goalkeeper present and goalkeeper not present) resulting in a total of 8 penalties being taken by each participant.

### Data analysis

Point of gaze data from the eye tracker was analysed offline using BeGaze (Ver. 3.4) software and was subject to frame by frame analysis. Each trial was tracked from the first frame the participant placed the football on the penalty spot up to the instance the ball was kicked (termed trial length). Areas of interest (AOI) were used to define key locations within the visual scene and comprised of; the football, goalkeeper, goal and other; “other” denotes fixations to task irrelevant locations within the display. To track point of gaze, a still image including all aforementioned AOI’s was loaded into the BeGaze software. Each point of gaze in the real-time dynamic visual scene was mapped manually (frame by frame) onto the AOIs in the still image. Each AOI was defined as an exact outline of the object shape.

Fixations were determined as four or more consecutive frames (≥120 ms) to an area of interest; a threshold consistent with previous research used to define a fixation (e.g. [5]).

The following variables were used to analyse eye tracking data;

#### Trial length

See description above.

#### Total fixation length on an AOI as a percentage trial length

Cumulative amount of time (milliseconds) the participant fixated at an AOI. Longer time spent fixating at a particular AOI allows more information to be obtained, indicating greater relevance to information processing and subsequent task execution [29]. Calculated as a percentage overall trial length to account for any differences in actual trial length between conditions.

#### Number of fixations within an AOI as a percentage of the total number of fixations within the trial

Higher number of fixations to a particular AOI provides an indication of the AOI’s relative relevance to information processing and subsequent task execution [29]. Calculated as a percentage overall trial length to account for any differences in actual trial length between conditions.

To provide additional information regarding the horizontal location where participants fixated in the goal, when participants fixated on the goal, analyses considered whether they fixated on the sides (left or right) of the goal. This, in combination with the goalkeeper AOI would provide an understanding of whether participants were fixating at the goalkeeper (centrally) or towards the peripheral areas of the goal during the penalty.

### Football end location

The bottom centre of the goal was defined as the ‘0′ horizontal coordinate. For the purpose of statistical analysis, to remove negative coordinates from the analysis, the absolute horizontal end ball location was used.

### Statistical analysis

Initial velocity of the football was analysed using a paired sample t-test (power vs. placement) with effect sizes calculated using Cohen’s d.

The number of goals scored and penalties kicked wide of the goal as a function of shot type was analysed using a Wilcoxon matched-pairs signed-ranks test. Success of penalty kicking performance was quantified using a goal/no goal coding strategy (1 indicating a goal scored, 2 indicating a miss or save). Number of penalties kicked wide of the goal; 1 indicating football landing within the goal (a goal or save), 2 indicating kicking wide of the goal.

A (separate) initial ANOVA was run on all dependent variables (excluding initial velocity and number of goals scored) and only revealed a main effect of repetition on trial length, indicating that the second penalty was executed quicker compared to the first penalty (p = .031). For this reason, data were averaged across repetition, subsequently resulting in a separate x2 shot (power, placement) x2 goalkeeper condition (goalkeeper present, goalkeeper not present) repeated measures ANOVA being used to analyse data. Level of significance was accepted at p<.05. Post hoc analyses, where appropriate, were performed using paired samples t-tests with a Bonferonni correction. Effect sizes were calculated using Partial Eta squared η_p_^2^.

## Results

### Penalty outcome

There was no significant effect of shot type on the number of goals scored (Z = −.632, p = .527) when comparing the power vs. placement penalty kick (mean 1.15 vs. 1.19, median 1.25 vs. 1.25 for power and placement penalties respectively). Analysis of the number of penalties scored/penalties missed between the goalkeeper and no goalkeeper condition was deemed redundant and was therefore excluded from the analysis.

There was no significant effect of shot type on the number of penalties that were kicked wide of the goal (Z = −.378, p = .705) when comparing the power vs. placement penalty kick (mean 1.25 vs. 1.29, median 1.25 vs. 1.50 for power and placement penalties respectively). Analysis of the number of penalties scored/penalties missed between the goalkeeper and no goalkeeper condition was deemed redundant and was therefore excluded from the analysis.

### Football velocity

Initial ball velocity was significantly higher in the power compared to the placement penalty (t (11) = −6.48, p<.001, *d* = −1.16). Group values for power and placement penalties were 19.1 (±2.4) m.s^−1^ vs. 16.5 (±2.1) m.s^−1^ respectively.

### Football end location

**Figure 1:**
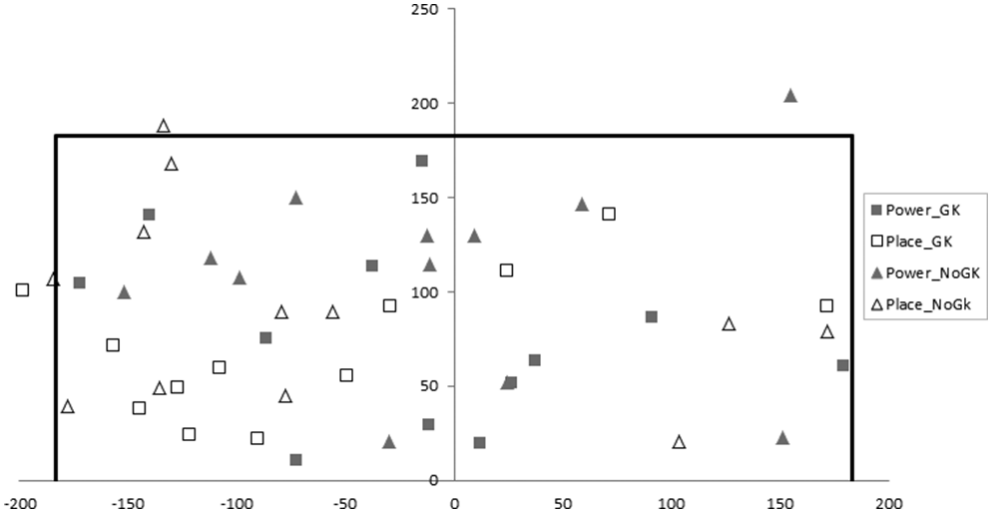
End point location of the football. Filled symbols denote power and unfilled placement penalties. Square symbols represent goalkeeper present and triangle no goalkeeper present conditions. Only the first penalty from each participant is presented for ease of graphical interpretation. NB. 0,0 coordinate represents the bottom middle of the goal. Units are measured in cm. Black outline represents the goal.

The football was kicked significantly more centrally in the goal when executing the power compared to placement penalty *F*(1,11) = 19.726, p<.001, η_p_^2^ = .642. There was no significant main effect of goalkeeper on football end location *F*(1,11) = .001, p = .973, η_p_^2^<.001, or was there a significant shot-by-goalkeeper interaction effect *F*(1,11) = .003, p = .954, η_p_^2^<.001. The average horizontal end ball location for each condition was 73±62 cm in the power and 128±48 cm in the placement penalty for the goalkeeper condition and 74±58 cm in the power and 127±41 cm in the placement penalty for the no goalkeeper condition (Fig. 1); 0 cm reflects the centre (middle) of the goal.

### Visual search parameters

#### Trial length

Trial length was significantly longer when taking the power vs. placement penalty kick *F*(1,11) = 8.276, p = .015, η_p_^2^ = .429 (5.21±1.34 sec vs. 4.82±1.37 sec respectively) and in the goalkeeper vs. no goalkeeper condition *F*(1,11) = 8.884, p = .013, η_p_^2^ = .447 (5.22±1.45 sec vs. 4.82±1.25 sec respectively). There was no significant shot-by-goalkeeper interaction effect *F*(1,11) = 2.010, p = 1.84, η_p_^2^ = .155.

#### Relative total fixation length

**Figure 2:**
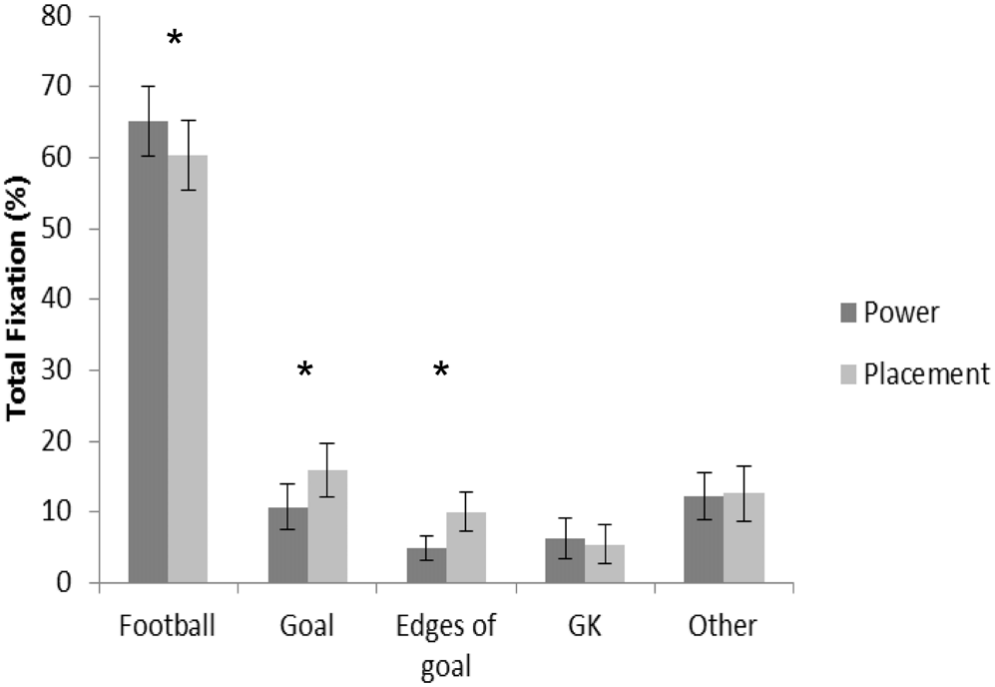
Percentage of time viewing specific areas of interest when taking the power and placement penalty (mean ± standard error). * denotes significant difference between condition. Data averaged across goalkeeper condition. Middle of goal also includes fixations on the goalkeeper (when present).

Total fixation length was significantly longer on the goal in the placement vs. power penalty *F*(1,11) = 6.115, p = .031, η_p_^2^ = .357 (15.95±12.89% vs. 10.72±10.91% respectively, Fig. 2) and in the no goalkeeper vs. goalkeeper condition *F*(1,11) = 14.271, p = .003, η_p_^2^ = .565 (18.95±12.56% vs. 7.72±8.76% respectively). There was no significant shot-by-goalkeeper interaction effect *F*(1,11) = .096, p = .762, η_p_^2^ = .009.

Total fixation length towards the sides of the goal was significantly longer in the placement vs. power shot *F*(1,11) = 5.131, p = .045, η_p_^2^ = .318 (10.03±9.54% vs. 4.83±5.84% respectively, Fig. 2) and in the no goalkeeper compared to goalkeeper condition *F*(1,11) = 5.082, p = .046, η_p_^2^ = .316 (9.38±8.78% vs. 5.48±7.35% respectively). There was no significant shot-by-goalkeeper interaction effect *F*(1,11) = .013, p = .910, η_p_^2^ = .001.

Total fixation length was significantly longer on the football in the power (65.13±16.97%) vs. placement penalty (60.34±17.26%) *F*(1,11) = 7.096, p = .022, η_p_^2^ = .392 (Fig. 2). No significant main effect was found for goalkeeper *F*(1,11) = 3.720, p = .08, η_p_^2^ = .253, or shot-by-goalkeeper interaction effect *F*(1,11) = .112, p = .744, η_p_^2^ = .010 (table 1).

**Table 1:** Visual search parameters as a function of goalkeeper and penalty condition.

	Goalkeeper (GK)		Penalty		
	GK present	GK not present	Power	Placement	Significant
Trial length (sec)	5.22 (1.45)	4.82 (1.25)	5.21 (1.34)	4.82 (1.37)	* ^Δ^
**Total fixation length (%)**					
Ball	60.55 (18.53)	64.92 (15.63)	65.13 (16.97)	60.34 (17.26)	*
GK	11.63 (11.13)	0 (0)	6.23 (10.04)	5.40 (9.57)	
goal	7.72 (8.76)	18.95 (12.56)	10.72 (10.91)	15.95 (12.89)	* ^Δ^
sides of goal	5.48 (7.35)	9.38 (8.78)	4.83 (5.84)	10.03 (9.54)	* ^Δ^
goal & gk	20.45 (12.92)	18.95 (12.56)	17.52 (11.50)	21.87 (13.56)	
other	13.72 (13.37)	11.10 (11.73)	12.23 (11.89)	12.59 (13.36)	
**No. fixations (%)**					
Ball	42.88 (17.06)	52.49 (18.28)	48.28 (19.44)	47.09 (17.14)	^Δ^
GK	13.72 (9.90)	0 (0)	7.81 (10.14)	5.91 (9.46)	*
goal	13.60 (12.61)	25.31 (14.86)	15.57 (15.22)	21.34 (14.53)	^Δ^
sides of goal	12.97 (12.64)	17.26 (15.42)	11.74 (12.27)	18.49 (15.27)	
goal & gk	21.96 (10.74)	25.31 (14.86)	23.85 (12.64)	23.42 (13.49)	
other	15.40 (13.59)	15.22 (13.65)	15.07 (13.29)	15.56 (14.03)	

*denotes shot and ^Δ^denotes goalkeeper main effect.

Nb. No statistical analysis was completed between fixation length or number between the goalkeeper and no goalkeeper condition for ‘GK’ AOIs as this comparison was deemed redundant.

When comparing the total fixation length on the goalkeeper, there was no significant main effect for shot *F*(1,11) = .455, p = .514, η_p_^2^ = .04, or shot-by-goalkeeper interaction effect *F*(1,11) = .455, p = .514, η_p_^2^ = .04 (table 1). Since the AOI goalkeeper was absent in the no goalkeeper condition, comparison between the goalkeeper and no goalkeeper condition was deemed redundant and was therefore excluded from the analysis.

When comparing the total fixation length on the goal and goalkeeper, there was no significant main effect of shot *F*(1,11) = 3.589, p = .085, η_p_^2^ = .246, goalkeeper *F*(1,11) = 1.036, p = .331, η_p_^2^ = .086, or shot-by-goalkeeper interaction effect *F*(1,11) = .083, p = .779, η_p_^2^ = .007 (table 1).

There was no significant main effect of total fixation length on other locations in shot *F*(1,11) = .034, p = .856, η_p_^2^ = .003, or goalkeeper condition *F*(1,11) = 1.844, p = .202, η_p_^2^ = .144, or was there a significant shot-by-goalkeeper interaction effect *F*(1,11) = .795, p = .392, η_p_^2^ = .067 (table 1).

#### Relative number of fixations

There were significantly more fixations on the goal in the no goalkeeper vs. goalkeeper condition *F*(1,11) = 13.088, p = .004, η_p_^2^ = .543 (25.31±14.86 vs. 13.60±12.61 respectively). No significant main effect was found for shot *F*(1,11) = 1.594, p = .233, η_p_^2^ = .127, or shot-by-goalkeeper interaction effect *F*(1,11) = .912, p = .360, η_p_^2^ = .077 (table 1).

Number of fixations in the sides of the goal was not affected by shot *F*(1,11) = 3.383, p = .09, η_p_^2^ = .235, goalkeeper *F*(1,11) = 2.325, p = .156, η_p_^2^ = .174, or significant shot-by-goalkeeper interaction effect *F*(1,11) = .041, p = .843, η_p_^2^ = .004 (table 1).

There were significantly more fixations on the football in the no goalkeeper vs. goalkeeper condition *F*(1,11) = 9.798, p = .010, η_p_^2^ = .471 (52.49±18.28 vs. 42.88±17.06 respectively). No significant main effect was found for shot *F*(1,11) = .119, p = .737, η_p_^2^ = .011, or shot-by-goalkeeper interaction effect *F*(1,11) = .879, p = .369, η_p_^2^ = .074 (table 1).

There were significantly more fixations on the goalkeeper in the power vs. placement penalty *F*(1,11) = 6.228, p = .03, η_p_^2^ = .362 (7.81±10.14 vs. 5.91±9.46% respectively)^2^. Since the AOI goalkeeper was absent in the no goalkeeper condition, comparison between the goalkeeper and no goalkeeper condition was deemed redundant and was therefore excluded from the analysis. There was no significant shot-by-goalkeeper interaction effect *F*(1,11) = 2.276, p = .160, η_p_^2^ = .171 (table 1).

Number of fixations on both the goalkeeper and goal was not affected by shot *F*(1,11) = .022, p = .884, η_p_^2^ = .002, goalkeeper *F*(1,11) = 1.524, p = .243, η_p_^2^ = .122, or shot-by-goalkeeper interaction effect *F*(1,11) = 3.088, p = .107, η_p_^2^ = .219 (table 1).

Number of fixations on other locations was not affected by shot *F*(1,11) = .054, p = .821, η_p_^2^ = .005, goalkeeper *F*(1,11) = .005, p = .942, η_p_^2^ = .000, or shot-by-goalkeeper interaction effect *F*(1,11) = .004, p = .951, η_p_^2^ = .000 (table 1).

## Discussion

When executing a soccer penalty kick there are two distinct approaches that can be adopted; a power penalty or a placement penalty. The aim of the current study was to investigate whether the type of penalty kick being taken influenced the visual search strategies adopted by the penalty kicker. The study also investigated whether the type of penalty kick affected football end location. Findings indicate key differences in visual search strategy and football end location as a function of type of penalty kick. During the power penalty, players fixated on the football for longer, fixated more frequently at the goalkeeper (and by implication at the centre of the goal), and kicked the football more centrally compared to the placement penalty. When executing the placement penalty, players fixated on the goal for longer, specifically the edges of the goal compared to the power penalty.

During the penalty kick, irrespective of condition, the football was the AOI fixated longest during the trial (∼60% of the trial, see table 1 and Fig. 2). This pattern of gaze behaviour has been similarly observed during penalties requiring a several step run up [11]. Whilst similar fixation durations on the football are not observed in penalties requiring a one-step run up (e.g. [27], [30], [31]), in the current study, this pattern of gaze behaviour is entirely expected due to the role that the eyes play in providing directional guidance to a target or object [32].

Compared to the placement penalty, when taking the power penalty the football was fixated significantly longer during the trial (see table 1 and Fig. 2). The increased time spent fixating on the football during the power penalty indicates a greater importance of obtaining visual information from the location of the football, which may be attributed to ensuring accurate foot-to-ball contact. Indeed, ball velocity is dependent upon the velocity of the foot at ball contact and the quality of foot-ball impact [33]–[36]. The additional time spent fixating on the football provided greater opportunity for adjustments in the movement execution during the approach to and execution of the kick, subsequently ensuring the quality of foot-ball contact. In combination with the above, it is relevant to note (albeit expected) that the football was kicked significantly harder when taking the power compared to placement penalty (19.5 m.s^−1^ vs 17.6 m.s^−1^ respectively). A subsidiary analysis correlating the time participants spent fixating the football and initial ball velocity during the power penalty provides some support for this discussion point. Despite observing a medium-strong effect size (r = .44), the association between the two variables was not significant (p = .155). The lack of significance may be attributed to a combination of a relatively low sample size and players in the group being of similar ability, resulting in ball velocities being clustered around a similar value (18–21 m.s^−1^ for power penalty). It is clear that further research is warranted to better understand the relationship between time spent fixating the football and the generation of ball velocity using a larger population group from a range of abilities.

The ball velocities recorded in the current study are similar to values reported in previous research which have ranged between 18–34 m.s^−1^ (see [37]). The average ball velocity during the power penalty is slightly lower than the threshold used by Kuhn [14] to quantify a power penalty (21 m.s^−1^) or the values reported by Lees and Nolan [35] or Neilson and Jones [37] for power penalties (25.45 m.s^−1^ and 27.05 m.s^−1^ respectively). A likely explanation for these differences is attributed to the level of performer and type of football. The aforementioned researchers recruited professional football players, whereas the current study recruited amateur and semi-professional football players. The current study used an indoor football, whereas the aforementioned researchers used an ‘outdoor’ football (see Lees and Nolan, [18] for a discussion on the individual characteristics of a football).

Whilst previous research has demonstrated that both power and placement penalties are regularly taken [14] and that penalties are frequently kicked to all locations within the goal [21], [23], no research has investigated how the end location of the penalty varies as a function of type of penalty kick. Results from the current study are the first to indicate that placement penalties are kicked wider in the goal compared to power penalties (128±48 cm vs. 73±62 cm respectively, see Fig. 1). This was accompanied by visual search data demonstrating that players fixated the peripheral areas of the goal for longer during the placement compared to the power penalty (see table 1, Fig. 2). These findings are consistent with previous research which has shown that when taking a placement penalty, players fixate and kick to the edges of the goal in an attempt to direct the ball to an area that the goalkeeper will struggle to reach/save [11], [13], [27], [31]. This visual search strategy supports previous research which has shown that the penalty taker aligns gaze with the desired end point location. When executing the power penalty, results from the football end location and visual search data suggest a different strategy being used to align gaze with the desired end point location. Despite the football being kicked more centrally when taking the power compared to the placement penalty, players fixated on the football and not the goalkeeper/centre of the goal for longer. This is contrary to the strategy adopted in the placement penalty. Importantly though, in the power penalty, players increased the number of fixations to the goalkeeper (and by implication the centre of the goal, see table 1). It is possible that players adopted a different visual search strategy to locate the centre of the goal to avoid sacrificing the time spent fixating on the football and compromising the quality of foot-ball contact required for generating ball velocity.

When executing the power penalty, it is unclear why participants kicked the ball centrally rather than to the corners of the goal. Previous research has shown that when executing movements at speed, accuracy decreases [16]. With less control between desired and actual football end location when taking the power penalty, it is possible that players kicked the ball centrally as a safety strategy to minimise the risk of kicking the ball wide of the goal [21]. With no significant difference between the number of penalties kicked wide of the goal in either power or placement condition, one could conclude that this strategy was successful. However, it is likely that this measure was not sensitive enough to identify subtle differences between desired and actual placement between the different types of penalty kick. In order to investigate any potential discrepancy between desired and actual end-point location as a function of type of penalty kick, future research should use a more sensitive measure requiring participants to identify the intended end location of the football prior to executing the penalty kick.

The presence of a goalkeeper can influence both visual search strategy [11], [12], [31] and football end location [10], [11]. For this reason, in the current study both power and placement penalties were taken with and without the presence of a goalkeeper. The consistency in results irrespective of goalkeeper condition demonstrates the robustness of the current findings. Rather surprisingly, where previous research has shown that the presence of a goalkeeper causes the football to be kicked more centrally in the goal (towards the goalkeeper, e.g. [10], [11]), this was not observed in the current study. The football end location remained the same irrespective of goalkeeper condition. A potential explanation for the differences between current and previous findings is provided through the response activation model and the saliency of the goalkeeper [38]. The response activation model suggests that prior to the execution of an action, fixating on both relevant and non-task relevant stimuli activate independent parallel action responses. Inhibitory processes are responsible for ensuring response tendencies to task irrelevant stimuli do not influence performance output. The ability of the inhibitory processes to ensure task irrelevant stimuli do not influence performance output is dependent upon the time prior to movement execution the stimuli is presented and the saliency of the object. Thus objects that are very salient will result in a response towards the object, whether intended or not [38].

Wood and Wilson [11] tasked football players with taking a penalty when facing a goalkeeper that was still or waving their arms during the kickers approach. The high degree of saliency from the goalkeeper (when waving their arms) resulted in both gaze behaviour and football end location being directed towards the centre of the goal/goalkeeper compared to when the goalkeeper remained still. Navarro et al. [10] compared penalty kicking performance in a goalkeeper vs. no goalkeeper paradigm, where the goalkeeper (when present) was only permitted to start moving in the ‘final portion’ of the kickers approach. This resulted in a less salient goalkeeper condition compared to that used by Wood and Wilson [11]. Despite Navarro et al [10] reporting the football being kicked more centrally in the goalkeeper compared to the no goalkeeper condition, there was no significant difference between these conditions. Significant differences in football end location were only observed in the no goalkeeper compared to goalkeeper condition when participants were required to inform the goalkeeper where they intended to kick the football prior to taking the penalty. Importantly, gaze behaviour was still directed towards the goalkeeper/middle of the goal. The goalkeeper condition used in the current study required the goalkeeper to remain still until the football was kicked, which subsequently resulted in a less salient goalkeeper than both aforementioned studies. In the present study, due to the relatively low degree of goalkeeper saliency, it is likely that players executing the penalty were able to develop effective inhibitory strategies to ensure performance outcome (football end location) was not compromised in the presence of the goalkeeper.

In either power or placement condition, there was no significant difference in the number of penalties scored (median 1.25 vs. 1.25 for power and placement penalties respectively), or the number of penalties kicked wide of the goal (median 1.25 vs. 1.50 for power and placement penalties respectively). One must be cautious in concluding that there is no superior penalty strategy to adopt in a ‘real’ football match to maximise the chance of scoring a penalty. In order to appropriately answer this particular research question (which was not the aim of the current study), further research is warranted using a full size goal with a wider range of participant (kicker and goalkeeper) expertise. It is unclear which type of penalty maximises the chance of scoring. It has been suggested that directing penalties to either top corner of the goal is the most successful goal scoring strategy [16], [39], however kicking to the centre of the goal may be more appropriate since goalkeepers frequently dive to the left or right, leaving the centre of the goal unattended and the chance of missing the goal entirely is minimal [40].

### Future work

Whilst the current research adds to our understanding of how penalty kicking technique influences football end location and visual search strategy, there are several relevant questions that remain unanswered. During a penalty kick, a complex relationship exists between penalty kicker and goalkeeper. Previous research has identified that the penalty kicker can deliberately miss-lead the goalkeeper in their indented placement of the penalty (opposite independent strategy [11]) and that the prior actions of the goalkeeper (e,g. distracting the kicker during their run up or even standing off-centre in the goal) can influence the area within the goal where the player kicks the ball [11], [28]. In the current study, instructions were developed for both the penalty taker and goalkeeper to ameliorate this complex relationship. However, future research should continue exploring this interaction. For example, it would be interesting to investigate whether prior information given to the penalty taker of where the goalkeeper typically dives within the goal influences where the ball is kicked.

### Summary

Findings from the present study highlight key differences in visual search strategy and football end location when executing a power vs. placement penalty kick. When executing the placement penalty, players fixated and kicked the football to the edges of the goal in an attempt to direct the ball to an area that the goalkeeper would have difficulty reaching and saving. When executing a power penalty, players fixated on the football for longer, fixated more frequently at the goalkeeper (and by implication the centre of the goal) and kicked the football more centrally. The increased time spent fixating the football during the power penalty is likely a strategy to ensure accurate foot-to-ball contact and subsequent generation of ball velocity. Aligning gaze and kicking the football centrally in the goal when executing the power compared to placement penalty may have been a strategy to reduce the risk of kicking wide of the goal altogether.

## Supporting Information

S1 File **Raw visual search data from participant group in each test condition.**
(ZIP)
